# Strength Tests and Numerical Simulations of Loess Modified by Desulfurization Ash and Fly Ash

**DOI:** 10.3390/ma15020512

**Published:** 2022-01-10

**Authors:** Zhi Cheng, Xinrong Cheng, Yuchao Xie, Zhe Ma, Yuhao Liu

**Affiliations:** 1 School of Science, North University of China, Taiyuan 030051, China; 18234162820@163.com (X.C.); mz605810831@163.com (Z.M.); liuyuhao327812@163.com (Y.L.); 2 Shanxi Road and Bridge Renewable Resources Development Co., Ltd., Taiyuan 030000, China; chaoyuxie@126.com

**Keywords:** desulfurization ash, modified loess, shear strength, FLAC3D, numerical simulation

## Abstract

Desulfurization ash and fly ash are solid wastes discharged from boilers of power plants. Their utilization rate is low, especially desulfurization ash, most of which is stored. In order to realize their resource utilization, they are used to modify loess in this paper. Nine group compaction tests and 32 group direct shear tests are done in order to explore the influence law of desulfurization ash and fly ash on the strength of the loess. Meanwhile, FLAC3D software is used to numerically simulate the direct shear test, and the simulation results and the test results are compared and analyzed. The results show that, with the increase of desulfurization ash’s amount, the shear strength of the modified loess increases first and then decreases. The loess modified by the fly ash has the same law with that of the desulfurization ash. The best mass ratio of modified loess is 80:20. When the mass ratio is 80:20, the shear strength of loess modified by the desulfurization ash is 12.74% higher than that of the pure loess on average and the shear strength of loess modified by fly ash is 3.59% higher than that of the pure loess on average. The effect of the desulfurization ash on modifying the loess is better than that of the fly ash. When the mass ratio is 80:20, the shear strength of loess modified by the desulfurization ash is 9.15% higher than that of the fly ash on average. Comparing the results of the simulation calculation with the actual test results, the increase rate of the shear stress of the FLAC3D simulation is larger than that of the actual test, and the simulated shear strength is about 8.21% higher than the test shear strength.

## 1. Introduction

Loess is widely distributed all over the world, including East Asia, Central Asia, Central Europe, the United States, Northern Russia, Alaska inland and South America [1]. About 10% of land area of the Earth is covered by loess [2]. In particular, about 6.6% of the land area is covered by loess in China [3]. Loess has the characteristics of collapsibility and high sensitivity. It may cause geological disasters such as landslides and uneven settlement of the foundation [2,4,5,6]. Therefore, the loess needs to be modified when building on it. There are some effective methods to modify loess. We can categorize them into two categories, which are physical methods and chemical methods. Strong compaction method, compact pile method and impact compaction method are common physical methods [7,8,9]. The chemical methods are the methods using solidified materials to react with the loess, thereby improving the strength, plasticity and permeability of the loess. At present, there are many kinds of solidified materials, including cement, lime, nano clay, lignin [4,10,11,12], etc.

Fly ash is a solid waste discharged by pulverized coal furnaces in power plants. As fly ash has good pozzolanic activity and certain self-hardening activity, it has been used widely in building material area. Comparing with cement and lime, the fly ash’s price is quite lower. Pei et al. studied the influence of lime and lime-fly ash on the strength and permeability of silty sand and silty clay loess in an engineering environment. The results show that, compared with results curing in laboratory conditions, the fast change periods of strength and permeability starts later and continues for a longer time when curing in an engineering environment. From 180 to 1090 days, the strength of lime-treated loess and lime-fly ash-treated loess increase by 13.2~16.5%, and their permeability coefficients decrease by 4~7.7% [13]. Phoak et al. studied the effects of fly ash on mechanical parameters of loess, and the results showed that fly ash can reduce the maximum dry density of loess, increase the optimal water content and improve the shear resistance [14]. Wang et al. studied the dynamic stress–strain relationship of saturated loess modified by fly ash in different amounts. It is proved that fly ash can improve the resistance of loess to liquefaction [15].

In recent years, as a clean and efficient coal-fired boiler, circulating fluidized bed (CFB) boiler has been widely used in thermal power plants. CFB boiler has the advantages of high desulfurization efficiency, low combustion temperature and low NOx emissions [16,17]. Same as the pulverized coal boiler, CFB boiler also discharges solid wastes. The solid wastes discharged from CFB boiler can be divided into two kinds, which are desulfurization ash and desulfurization slag. Among them, desulfurization ash is collected in the gas flue, and desulfurization slag is discharged from the bottom of the furnace [18]. At present, the utilization rate of desulphurization ash and slag is less than 10%, and most of them are in the state of stockpiling. The utilization way urgently needs to broaden out [19,20,21]. The same as fly ash, the desulfurization ash also contains a large amount of SiO_2_ and Al_2_O_3_, it also has good pozzolanic activity. Therefore, it has the material basis for use as building materials. However, different from fly ash, the desulfurization ash contains f-CaO and SO_3_, it has expansibility, which limits its large-scale use [22,23]. Researchers try to use desulfurization ash as cement or concrete additives [20], geopolymers [23], low-strength engineering backfill materials [21], etc. However, there are few reports on using desulfurization ash to improve loess.

In this paper, the influence laws of desulfurization ash and fly ash on the loess were investigated. Compaction tests and direct shear tests were performed to research the shear strength. The effects of desulfurization ash and fly ash on the shear strength were analyzed and compared. FLAC3D numerical software was used to simulate the loess modified by desulfurization ash, and the simulation results were compared with the experimental results. The research results can provide a theoretical basis of the loess modified by desulfurization ash and fly ash for the engineering application, and provide a utilization method for desulfurization ash and fly ash.

## 2. Materials and Methods

### 2.1. Raw Materials

**Figure 1:**
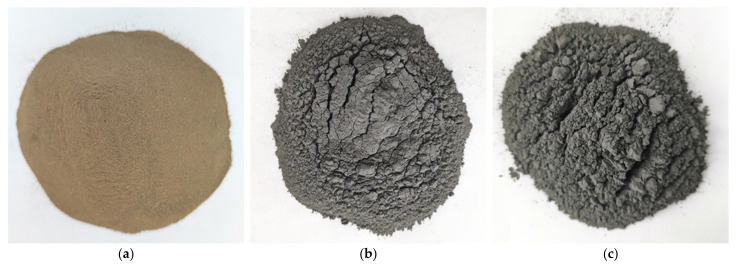
Digital images: (**a**) loess; (**b**) desulfurization ash; and (**c**) fly ash.

**Figure 2:**
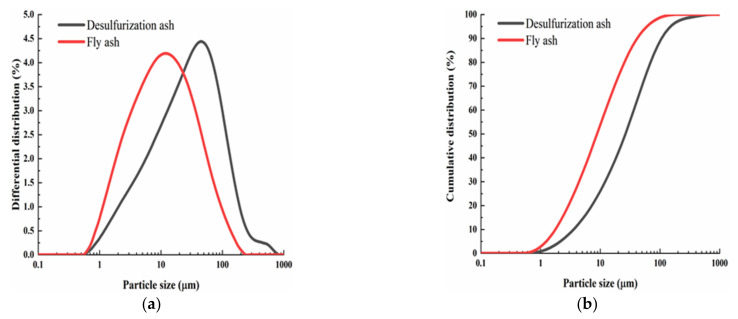
Particle size distribution curves: (**a**) differential particle size distribution curves; (**b**) cumulative particle size distribution curves.

The images of the loess, the desulfurization ash and the fly ash used in the experiment are shown in Figure 1. The loess was collected from Jiancaoping District, Taiyuan City (Taiyuan, China), and its main physical properties are shown in Table 1. The desulfurization ash was provided by Shanxi Pingshuo Coal Gangue Power Plant (Shouzhou, Shanxi, China). Its color is gray, and the density is 2240 kg/m^3^. Its chemical composition is shown in Table 2. The fly ash is an ordinary grade II fly ash from Taiyuan No. 2 Thermal Power Plant (Taiyuan, Shanxi, China). It can be seen from Figure 1 that the fly ash is darker than the desulfurization ash. The particle size of fly ash varies between 0.5–300 μm, most of which are less than 75 μm, the specific surface area is 361 m^2^/kg, the density is 2330 kg/cm^3^, and its specific chemical composition is shown in Table 3 [24]. The particle size distribution curves of desulfurization ash and fly ash are shown in Figure 2.

### 2.2. Specimen Preparation

At the beginning of the experiment, the loess was dried to constant mass in an oven and passed through a sieve with an aperture of 2 mm. Then, it was sealed and stored in a plastic box for later use. The desulfurization ash and the fly ash were also dried and passed through a sieve with an aperture of 80 μm. The loess and the desulfurization ash were mixed with water according to Table 4. The water was put into a small spray can and sprayed slowly, and the mixture was stirred to ensure the loess and the desulfurization ash evenly distributed. After the specimens were produced, they were put into a plastic bag and weighed. The number and moisture content of each specimen were labeled. During the curing process, the specimen was in a free state. The moisture content was re-measured before the compaction tests and the direct shear tests. The moisture content deviation was within the range of ω = ±0.2%. The same method was used to prepare the specimens of loess modified by fly ash [25].

### 2.3. Compaction Tests

The compaction test scheme is shown in Table 4. Nine groups of specimens were designed with different mass ratios of desulfurization ash, fly ash and the loess. The compaction tests were carried out according to the “Standard for Geotechnical Test Methods” (GB/T 50123-2019), and the maximum dry density and optimal moisture content of each group were obtained [25,26].

### 2.4. Direct Shear Tests

The direct shear test scheme is shown in Table 5. A total of 32 groups of specimens were designed with different mass ratios and different normal stresses. The ZJ series strain control direct shear instrument was used in the tests, which was produced by Nanjing Loess Instrument Factory Co., LTD (Najing, China). The direct shear tests were carried out according to GB/T 50123-2019, the same standard with the compaction tests. Under undrained and unconsolidated conditions, the specimens were tested until destroyed at a shear rate of 0.8–1.2 mm/min under different vertical pressures (50 kPa, 100 kPa, 200 kPa, and 300 kPa). The shear parameters of the specimens were obtained by the direct shear tests [25].

## 3. Results and Discussion

### 3.1. Effect of Different Solidified Materials on Dry Density and Moisture Content of the Loess

**Figure 3:**
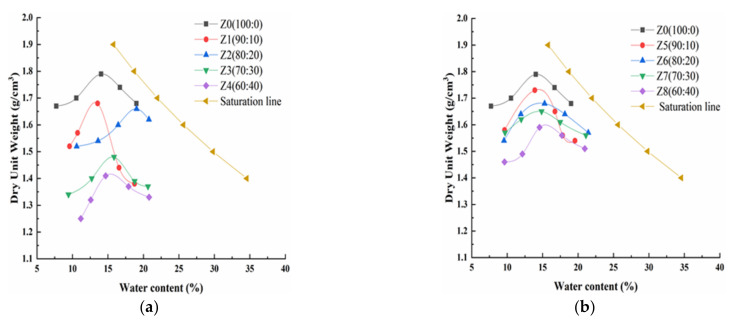
Compaction curves of the loess modified by different solidified materials. (**a**) Desulfurization ash; and (**b**) fly ash.

**Figure 4:**
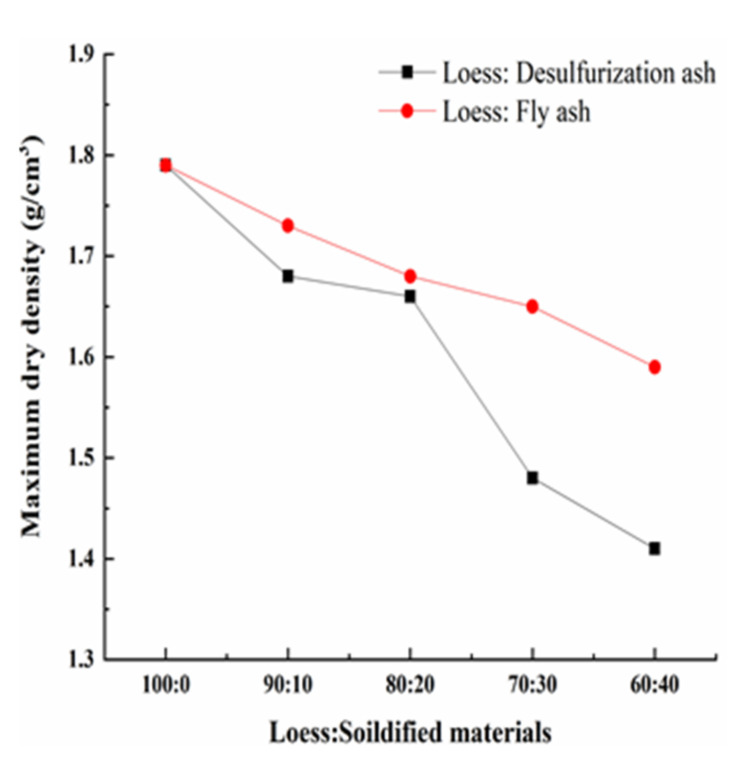
The relationship between maximum dry density and solidified materials.

**Figure 5:**
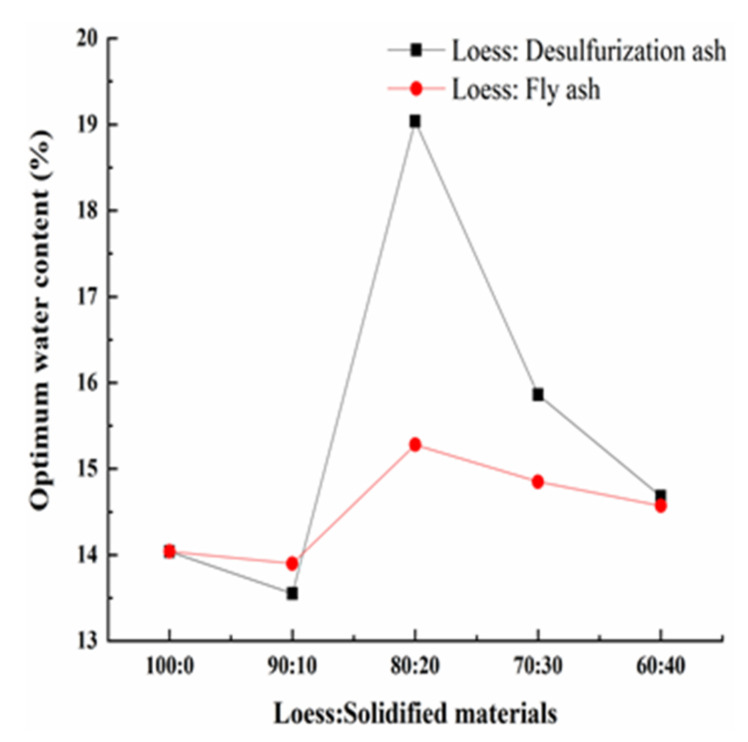
The relationship between optimal moisture content and solidified materials.

The relationship between moisture content and dry density of each specimen was obtained by the compaction tests. The compaction curves of modified loess with different solidified materials are shown in Figure 3. The maximum dry density and optimal water content of each specimen can be obtained according to Figure 3. Then, the relationship between maximum dry density and loess modified by different solidified materials was obtained, and the relationship between the maximum water content and loess modified by different solidified materials was obtained as well. They are shown in Figure 4 and Figure 5 [26].

Figure 4 shows that the maximum dry density of the modified loess decreases gradually as the amount of the solidified materials increases when the solidified materials replace the loess in equal quantities. On one hand, as the density of the solidified material is less than that of loess, the overall of the mixture’s density decreases. On the other hand, as the desulfurization ash and the fly ash both have pozzolanic activity and self-hardening activity, the production of the hydration reaction and the pozzolanic reaction will occur, which will increase the solidified cohesion and decrease the particle size of the loess. The greater the solidified material’s amount, the lower the maximum dry density. Figure 4 also shows that the maximum dry density of the loess modified by the desulfurization ash is less than the fly ash under the same mass ratio. That is because that the density of the desulfurization ash is lower than that of the fly ash.

Figure 5 shows that as the amount of the solidified material increases, the optimal moisture content first increases, then decreases and lastly increases. When the mass ratio of the loess and the solidified material is 80:20, the optimal moisture content reaches the maximum. The value of the loess modified by the desulfurization ash is much bigger than that of the loess modified by the fly ash. On one hand, the microstructure of the two solidified materials is different. The desulfurization ash is coarse, irregular, loose and porous, while the fly ash is spheroidal and smooth. The desulfurization ash would absorb more water than the fly ash. On the other hand, the chemical composition is also different. The desulfurization ash has higher content of calcium oxide than the fly ash. Meanwhile, the desulfurization ash has anhydrite, which does not exist in the fly ash. The chemical reaction of the desulfurization ash needs more water [18,27].

### 3.2. Effect of Different Solidified Materials on Shear Strength of the Loess

**Figure 6:**
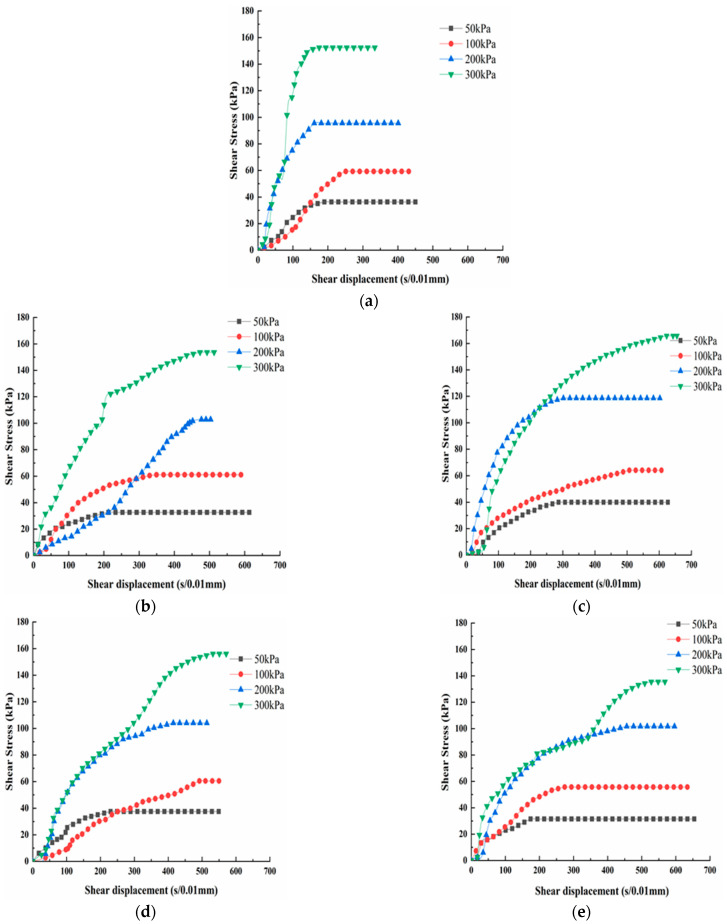

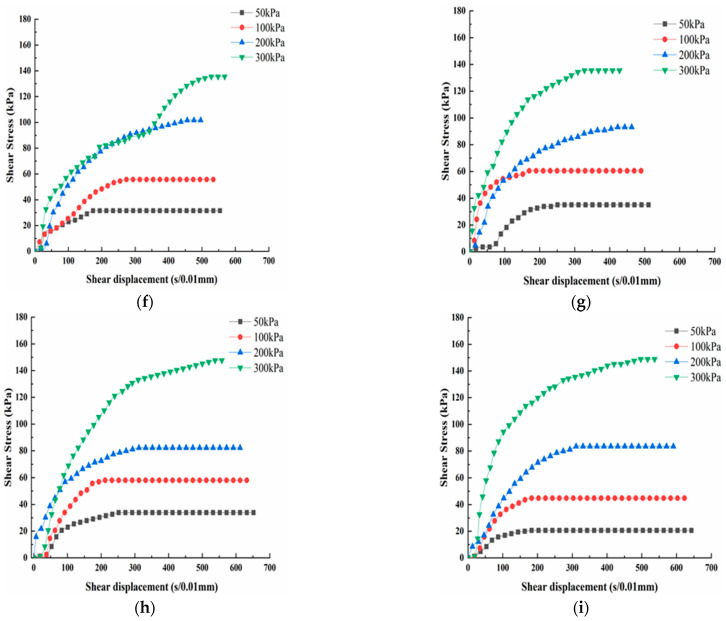
The stress-displacement curves of the modified loess. (**a**) Z0; (**b**) Z1; (**c**) Z2; (**d**) Z3; (**e**) Z4; (**f**) Z5; (**g**) Z6; (**h**) Z7; and (**i**) Z8.

The stress-displacement curves of the modified loess are shown in Figure 6. The curves can be categorized into three stages. In the first stage, the shear stress increases rapidly, while the shear displacement has little increase. In the second stage, the stress increases at a slower rate and reaches the shear peak. In the last stage, when the displacement increases, the stress no longer increases, so the curve tends to be stable. The stress-displacement curves under different normal stresses have the same law. The bigger the normal stress, the bigger the ultimate shear stress.

**Figure 7:**
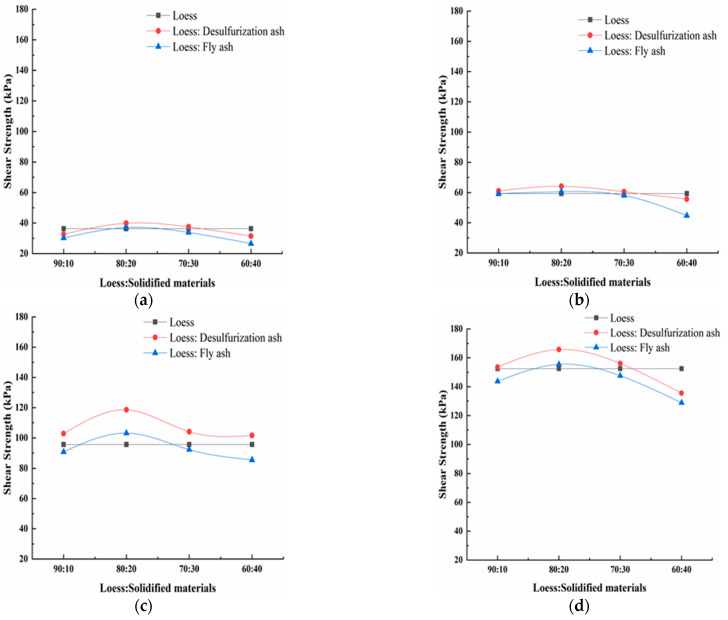
The shear strength of the modified loess under different normal stresses. (**a**) 50 kPa; (**b**) 100 kPa; (**c**) 200 kPa; and (**d**) 300 kPa.

The shear strength of the modified loess under different normal stresses is shown in Figure 7. From Figure 7, it can be seen that the shear strength of the loess modified by the desulfurization ash has the same law as that of the loess modified by fly ash under a different mass ratio. With the amount of the modified material increasing, the shear strength first increases, and then decreases. When the mass ratio is 80:20, the shear strength reaches the maximum. When the mass ratio is 90:10, 70:30 and 60:40, the shear strength of the modified loess has little increase or even decreases compared with the pure loess. When the normal stress is 200 kPa and the mass ratio is 80:20, the shear strength of the loess modified by the desulfurization ash is about 24.05% bigger than that of the pure loess, and the shear strength of the loess modified by the fly ash is about 7.93% bigger than that of the pure loess. When the mass ratio is 80:20, the shear strength of the loess modified by the desulfurization ash is about 12.74% bigger than that of the pure loess on average, and the shear strength of the loess modified by the fly ash is about 3.59% bigger than that of the pure loess on average. The best mass ratio of modified loess is 80:20.

Under the same mass ratio, the shear strength of the loess modified by desulfurization ash is bigger than that of the loess modified by fly ash. When the mass ratio is 80:20, the shear strength of the loess modified by the desulfurization ash is about 9.15% bigger than that of the fly ash on average. When the normal stress is 200 kPa and the mass ratio is 80:20, the shear strength has the maximum difference. The shear strength of the loess modified by the desulfurization ash is about 16.12% bigger than that of the fly ash. The desulfurization ash has a better effect than the fly ash on modifying the loess. Samnang Phoak et al. found that fly ash can increase the California bearing ratio (CBR) value of loess, but it is not obvious [27,28,29].

As with fly ash, desulfurization ash contains silicon oxide (SiO_2_) and aluminium oxide (Al_2_O_3_). However, unlike fly ash, the desulfurization ash contains more calcium oxide (CaO) and anhydrite (II -CaSO_4_). The CaO can react with the SiO_2_ to form hydrated calcium silicate (C-S-H) gel, and react with the Al_2_O_3_ to form calcium aluminate hydrate (C-A-H) gel, both of which have hydraulicity and can produce strength. The II -CaSO_4_ cannot directly participate in the hydration reaction. It needs to dissolve to gypsum first. Then, the gypsum reacts with calcium aluminate hydrate (C-A-H) to form high sulfur calcium sulphoaluminate hydrate, commonly known as ettringite (AFt). The AFt is irregular in the shape of a hexagonal needle column. As the reaction continues, the AFt can develop into a spatial structure, closely connect with the loess particles, and help the modified loess to gain a relatively high strength in the early stage [27,28].

### 3.3. Effect of Different Solidified Materials on the Strength Parameters of the Loess

**Figure 8:**
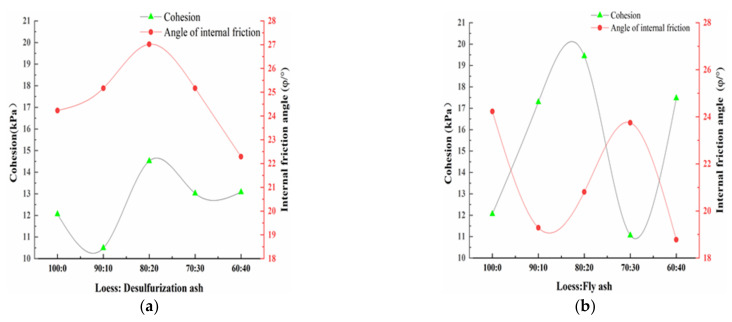
Curves of strength parameters of the loess modified by different solidified materials. (**a**) Desulfurization ash; and (**b**) fly ash.

The curves of the strength parameters of the modified loess are shown in Figure 8.

Figure 8 shows the change law of the cohesion and the internal friction angle under different mass ratio. It can be seen from Figure 8a that when the amount of the desulfurization ash increases from 0 to 20%, the internal friction angle and the cohesion of the modified loess both increase. When the amount of the desulfurization ash increases over 20%, the internal friction angle and the cohesion of the modified loess begin to decrease. Therefore, it can be concluded that the best amount of the desulfurization ash is 20%, and the best mass ratio of the loess and the desulfurization ash is 80:20. As opposed to cohesion, the internal friction angle plays a bigger role. It can also be seen from Figure 8b that when the amount of the fly ash increases, the internal friction angle of the modified loess fluctuates up and down, all are inferior to the pure loess. It can be concluded that adding fly ash cannot raise the friction of the loess. When the amount of the fly ash increases from 0 to 20%, the cohesion of the modified loess obviously increases. When the mass ratio is 80:20, the cohesion of the modified loess reaches the maximum. As opposed to internal friction angle, the cohesion plays a bigger role. The influence of fly ash on the modified loess’s internal friction angle and cohesion shows similar laws with magnesium oxide and cement [12,14,30].

The influence mechanism of the desulfurization ash and fly ash on loess is quite different. On one hand, the desulfurization ash has irregular microstructure and rough surface. When mixed with loess, it can increase the contact surface with loess, and fill well with loess. The friction between the modified loess particles can be raised. While the fly ash has a rounded microstructure and smooth surface, it cannot make the modified loess particles contact closely. Therefore, the friction of the modified loess cannot be raised. On the other hand, the desulfurization ash has anhydrite (II-CaSO_4_), which needs to dissolve to gypsum first, and then reacts with calcium aluminate hydrate (C-A-H) to form ettringite (AFt). The AFt is a hexagonal needle column crystal, and grows disorderly and irregularly. It has a good effect to link the modified loess as a whole. In general, desulfurization ash is better than fly ash to modify the loess [18,22,23].

### 3.4. FLAC3D Numerical Simulation

**Figure 9:**
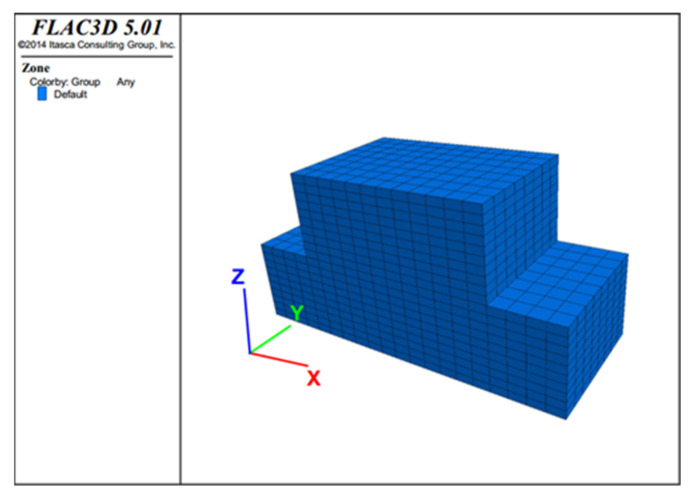
Direct shear test model diagram.

The numerical simulation of the direct shear test was carried out on the loess modified by the desulfurization ash as the mass ratio is 80:20. It is assumed that the mixture of desulfurization ash and loess is homogeneous. Two separate hexahedral loess blocks are established. The upper hexahedral loess block is length = 18 mm, width = 10 mm, height = 5 mm, and the lower hexahedral loess block is length = 20 mm, width = 10 mm, height = 5 mm. The upper block can move freely while the lower block is fixed. The model has a total of 3800 units. The interface of the lower loess block (Z = 5) is the shear plane, which properties are defined. In numerical simulations, the Mohr-Coulomb model was used. The specific parameters are shown in Table 6. The model is established, as shown in Figure 9. The model can move freely in the X direction, and the constant velocity is set at 8.0 × 10^−6^ m/step. The vertical downward normal stress is applied to the Z = 10 mm surface, and the values are 50 kPa, 100 kPa, 200 kPa and 300 kPa.

**Figure 10:**
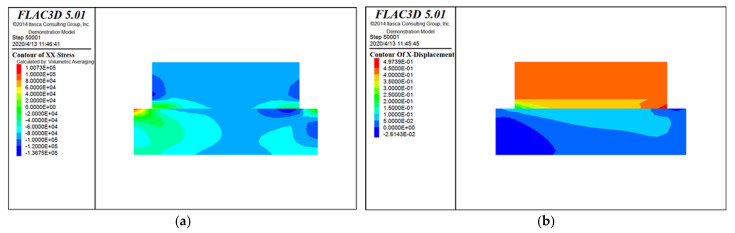
*X*-direction stress and displacement cloud diagram. (**a**) Stress cloud diagram; and (**b**) displacement cloud diagram.

**Figure 11:**
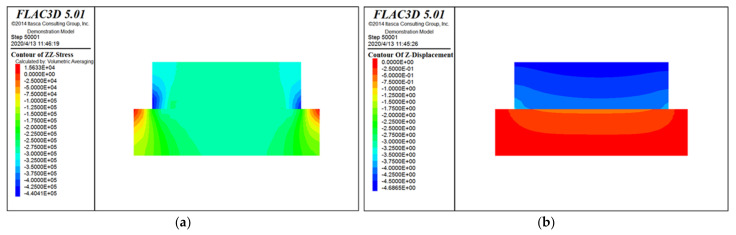
*Z*-direction stress and displacement cloud diagram. (**a**) Stress cloud diagram; and (**b**) displacement cloud diagram.

**Figure 12:**
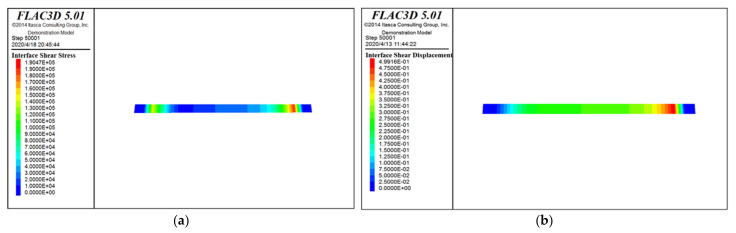
Stress and displacement cloud diagram on the shear surface. (**a**) Stress cloud diagram; and (**b**) displacement cloud diagram.

The specific simulation calculation cloud diagram is shown as Figure 10, Figure 11 and Figure 12. Figure 10 is the stress and displacement cloud diagram when the horizontal stress is applied to the modified loess in the *X* direction. Figure 11 is the stress and displacement cloud diagram when a normal stress is applied to the modified loess in the *Z* direction. Figure 12 is the stress and displacement cloud diagram of the stress cloud on the shear surface.

From Figure 12, it can be seen that, affected by the loess’s own weight and the vertical stress applied to the loess, after the horizontal stress is applied, the main part of the loess is concentrated on the edge of the contact plane, and the force from both sides to the middle gradually weakens. On both sides of the lower loess block, the force is obviously restricted by the boundary conditions. The displacement of the upper part of the loess is the largest, and the displacement at the shear surface is of a diffusion type, which is in line with the actual situation, indicating that the calculation result is reliable.

**Figure 13:**
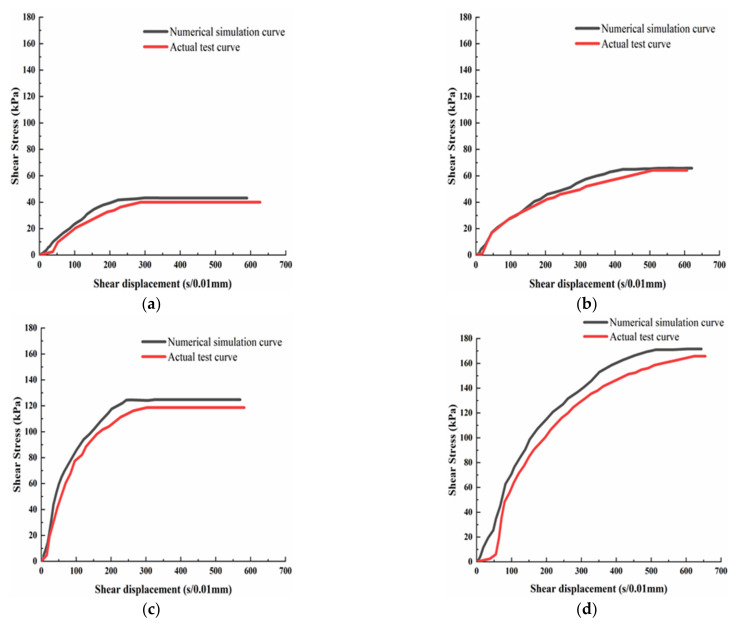
The shear stress-displacement contrast curves: (**a**) 50 kPa; (**b**) 100 kPa; (**c**) 200 kPa; and (**d**) 300 kPa.

The shear stress-displacement curves under four different normal stresses (50 kPa, 100 kPa, 200 kPa, and 300 kPa) are drawn according to the direct shear test results and numerical calculation results, which are shown in Figure 13.

From Figure 13, it can be seen that the increase rate of the shear stress of the FLAC3D simulation is larger than that of the test when the shear displacement increases. The loess’s rigidity of the simulation is bigger than that of the test. Compared with the test results, the simulated shear strength is about 8.21% higher. In the simulation calculation process, all conditions are idealized. The mixture of loess and desulfurization ash is regarded as a homogeneous material, the influence of drainage conditions is ignored, and error influence in the actual test process is not considered. As a result, the simulation results are overall higher than the test results.

## 4. Conclusions

The shear strength of the loess modified by desulfurization ash and fly ash are researched in the paper. Nine group compaction tests, 32 group direct shear tests and FLAC3D numerical simulation are done. Several conclusions can be drawn as follows:

1. As the amount of the solidified materials increases, the maximum dry density of the modified loess decreases. Under the same mass ratio of the loess to the solidified material, the maximum dry density of the loess modified by the desulfurization ash is less than that of the fly ash. When the mass ratio is 80:20, the optimal moisture content reaches the maximum. The value of the loess modified by the desulfurization ash is much bigger than that of the loess modified by the fly ash.
2. With the amount of the modified material increasing, the shear strength first increases, and then decreases. The best mass ratio of modified loess is 80:20. When the mass ratio is 80:20, the shear strength reaches the maximum, the shear strength of the loess modified by the desulfurization ash is about 12.74% bigger than that of the pure loess on average, and the shear strength of the loess modified by the fly ash is about 3.59% bigger than that of the pure loess on average. The desulfurization ash has a better effect than the fly ash on modifying the loess.
3. When the desulfurization ash is used to modify the loess, the internal friction angle plays a bigger role as opposed to cohesion. While the fly ash is used to modify the loess, the cohesion plays a bigger role as opposed to internal friction angle. This is due to the differences of their microstructures, the surface features and the chemical compositions.
4. Using FLAC3D to simulate the direct shear test is feasible. However, there are still some differences. The increase rate of the shear stress of the simulation is larger than that of the actual test when the shear displacement increases, and the simulated shear strength is about 8.21% higher than the tested shear strength.

## Figures and Tables

**Table 1:** Main physical properties of the loess.

Natural Moisture Content (%)	Dry Density (g/cm^3^)	Liquid Limit (%)	Plastic Limit (%)	Plastic Index I_p_ (%)	Specific Gravity G_s_
13.2	1.74	25.1	14.3	10.8	2.71

**Table 2:** Main chemical composition of desulfurization ash (%).

SiO_2_	Al_2_O_3_	CaO	SO_3_	Fe_2_O_3_	MgO	K_2_O	P_2_O_5_	Na_2_O
42.19	25.9	10.99	5.91	3.1	1.35	0.79	0.12	0.06

**Table 3:** Main chemical composition of fly ash (%).

SiO_2_	Al_2_O_3_	Fe_2_O_3_	CaO	K_2_O	TiO_2_	MgO	Na_2_O	SO_3_	P_2_O_5_	NiO
53.97	31.15	4.16	4.01	2.04	1.13	1.01	0.89	0.73	0.67	0.14

**Table 4:** Compaction test scheme.

Group Number	Solidified Materials	Loess: Solidified Materials
Z0	-	100:0
Z1	Desulfurization ash	90:10
Z2	Desulfurization ash	80:20
Z3	Desulfurization ash	70:30
Z4	Desulfurization ash	60:40
Z5	Fly ash	90:10
Z6	Fly ash	80:20
Z7	Fly ash	70:30
Z8	Fly ash	60:40

**Table 5:** Direct shear test scheme.

Solidified Materials	Loess: Solidified Materials	Normal Stresses (kPa)	Total Numbers of Specimen
Desulfurization ash	90:10, 80:20, 70:30, 60:40	50, 100, 200, 300	16
Fly ash	90:10, 80:20, 70:30, 60:40	50, 100, 200, 300	16

**Table 6:** Main parameters.

No.	Normal Stress (kPa)	Normal Stiffness (MN/m)	Tangential Stiffness (MN/m)	Internal Friction Angle	Dilatancy Angle	Cohesion (kPa)	Dilatancy Angle (Mpa)	Volume Modulus (Mpa)
1	50	9	9	27.06	5	14.52	0.8	1.3
2	100	9	9	27.18	5	14.57	1.2	1.5
3	200	9	9	27.32	5	14.65	1.5	1.8
4	300	9	9	27.41	5	14.53	1.7	2.1

## Data Availability

The data presented in this study are available on request from the corresponding author.
